# Protein Expression Profiling of Giant Cell Tumors of Bone Treated with Denosumab

**DOI:** 10.1371/journal.pone.0148401

**Published:** 2016-02-10

**Authors:** Kenta Mukaihara, Yoshiyuki Suehara, Shinji Kohsaka, Keisuke Akaike, Yu Tanabe, Daisuke Kubota, Midori Ishii, Tsutomu Fujimura, Saiko Kazuno, Taketo Okubo, Tatsuya Takagi, Takashi Yao, Kazuo Kaneko, Tsuyoshi Saito

**Affiliations:** 1 Department of Orthopedic Surgery, Juntendo University School of Medicine, Tokyo, Japan; 2 Department of Pathology, Memorial Sloan-Kettering Cancer Center, New York, New York, United States of America; 3 Laboratory of Biochemical Analysis, Central Laboratory of Medical Sciences, Juntendo University School of Medicine, Tokyo, Japan; 4 Department of Human Pathology, Juntendo University School of Medicine, Tokyo, Japan; Faculté de médecine de Nantes, FRANCE

## Abstract

Giant cell tumors of bone (GCTB) are locally aggressive osteolytic bone tumors. Recently, some clinical trials have shown that denosumab is a novel and effective therapeutic option for aggressive and recurrent GCTB. This study was performed to investigate the molecular mechanism underlying the therapeutic effect of denosumab. Comparative proteomic analyses were performed using GCTB samples which were taken before and after denosumab treatment. Each expression profile was analyzed using the software program to further understand the affected biological network. One of identified proteins was further evaluated by gelatin zymography and an immunohistochemical analysis. We identified 13 consistently upregulated proteins and 19 consistently downregulated proteins in the pre- and post-denosumab samples. Using these profiles, the software program identified molecular interactions between the differentially expressed proteins that were indirectly involved in the RANK/RANKL pathway and in several non-canonical subpathways including the Matrix metalloproteinase pathway. The data analysis also suggested that the identified proteins play a critical functional role in the osteolytic process of GCTB. Among the most downregulated proteins, the activity of MMP-9 was significantly decreased in the denosumab-treated samples, although the residual stromal cells were found to express MMP-9 by an immunohistochemical analysis. The expression level of MMP-9 in the primary GCTB samples was not correlated with any clinicopathological factors, including patient outcomes. Although the replacement of tumors by fibro-osseous tissue or the diminishment of osteoclast-like giant cells have been shown as therapeutic effects of denosumab, the residual tumor after denosumab treatment, which is composed of only stromal cells, might be capable of causing bone destruction; thus the therapeutic application of denosumab would be still necessary for these lesions. We believe that the protein expression patterns and the results of the network analysis will provide a better understanding of the effects of denosumab administration in patients with GCTB.

## Introduction

Giant cell tumors of bone (GCTB) are rare benign, but locally aggressive lesions that are associated with significant bone destruction and soft tissue extension [1]. The rate of local recurrence following surgical curettage is relatively high at approximately 25% [2]. In rare cases, GCTB may metastasize to the lung and malignant changes may occur [3].

Histologically, GCTB is composed of three main cellular components of mesenchymal fibroblast-like stromal cells which highly express the receptor activator of nuclear factor kappa-B ligand (RANKL); some of the rounded mononuclear cells and osteoclast-like multinucleated giant cells express RANK [4–6]. These cellular components interact with various factors and play a significant role in the osteolytic process, leading to bone destruction. In the presence of RANKL and macrophage colony- stimulating factor (C-FMS) acting as co-factor, RANK mediates osteoclast formation by enhancing the expression of enzymes that degrade the various components of bone [7, 8]. Simultaneously, endogenous osteoprotegerin (OPG) inhibits both differentiation and function of osteoclasts by competing for and neutralizing RANKL [9].

A RANKL inhibitor, Denosumab, has recently been developed. Some studies have reported that this agent is a novel and effective treatment option for cases of aggressive GCTB [10]. Denosumab exerts its effects by binding to RANKL, inhibiting bone destruction and eliminating giant cells [11, 12]. These studies indicate that denosumab plays a critical role in the therapeutic strategy for GCTB. However, this mechanism alone is not enough to explain the potent effects of the therapeutic application of denosumab. Further unknown effects might be involved in the underlying processes [13]. Despite its basic and clinical significance, the mechanisms involved in the efficacy of denosumab treatment remain to be fully elucidated. However, a few studies have investigated the associations between GCTB and RANKL/RANK signaling [11, 12].

Among the various “-omics” approaches using clinical specimens to understand the molecular basis underlying disease formation [14], the proteomics approach is helpful as it allows further insight into the biology of GCTB treated with denosumab because proteomic studies can identify the differences in molecular expression and pathway dysregulation that occur in response to different treatments [9]. A deeper understanding of the proteomes within the framework of the RANK/RANKL pathway (which is involved in the therapeutic effects of Denosumab) would provide us with a better therapeutic strategy for GCTB. At present, however, the protein expression profile changes that occur in GCTB patients who receive denosumab remain to be elucidated.

In this study, to identify the proteins in GCTB patients that are significantly affected denosumab, we conducted proteomic studies using clinical samples obtained from GCTB patients before and after denosumab treatment. Furthermore, to further the understanding of these biological networks, we performed an Ingenuity Pathway Analysis (IPA) using these protein profiles. Our results indicated that MMP-9 activity might play a critical role in the promotion of tumor invasiveness in GCTB patients who are treated with denosumab.

## Materials and Methods

**Fig 1:**
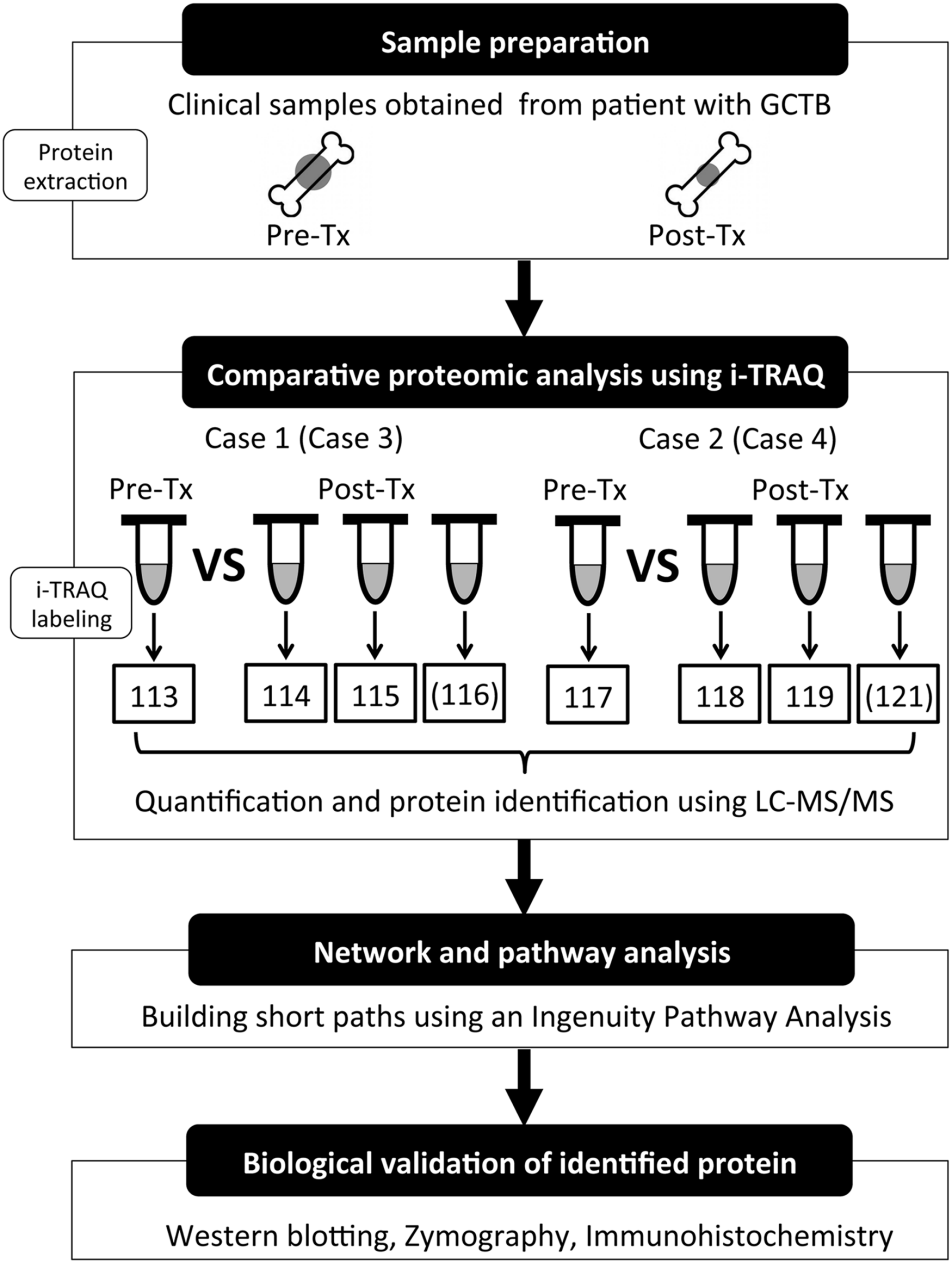
The work flow for the identification of the novel proteins dysregulated by denosumab treatment in GCTB. The protein was extracted from each of the resected samples as a lysate, then digested using trypsin to generate a proteolytic peptide. Each peptide was labelled with iTRAQ Tags (113, 114, 115, 116, 117, 118, 119 and 121) for the eight-plex iTRAQ analysis. These labelled and combined peptides were analyzed by LC-MS/MS for the purposes of identification and quantification. Using the obtained dataset, the molecular networks and pathways of the differentially expressed proteins were subjected to an IPA. Further biological validation was obtained by Western blotting, zymography and immunohistochemistry.

This study work flow is shown in Fig 1.

### Patients and tumor samples

Four GCTB patients were treated with denosumab at the department of Orthopaedic Surgery in Juntendo University Hospital in Tokyo, Japan. We collected 14 samples from the 4 patients (1 pre-treatment sample and 2 or 3 post-treatment samples). We divided the 4 cases into two groups, including the 1st set (test set; cases 1 and 2) and the 2nd set (validation set; cases 3 and 4). The clinicopathological features of these 4 cases are shown in Table 1.

**Table 1:** Clinicopathological characteristics of patients of GCTB treated with denosumab.

Case No.	Age	Sex	Location	Dose of denosumab	Primary or Reccurrence	Surgical treatment
1	50	F	Femur	120 mg (× 3)	Primary	Wide resection
2	20	M	Femur	120 mg (× 3)	Primary	Curratage
3	45	M	Femur	120 mg (× 3)	Primary	Wide resection
4	32	F	Fibula	120 mg (× 3)	Primary	Wide resection

**Fig 2:**
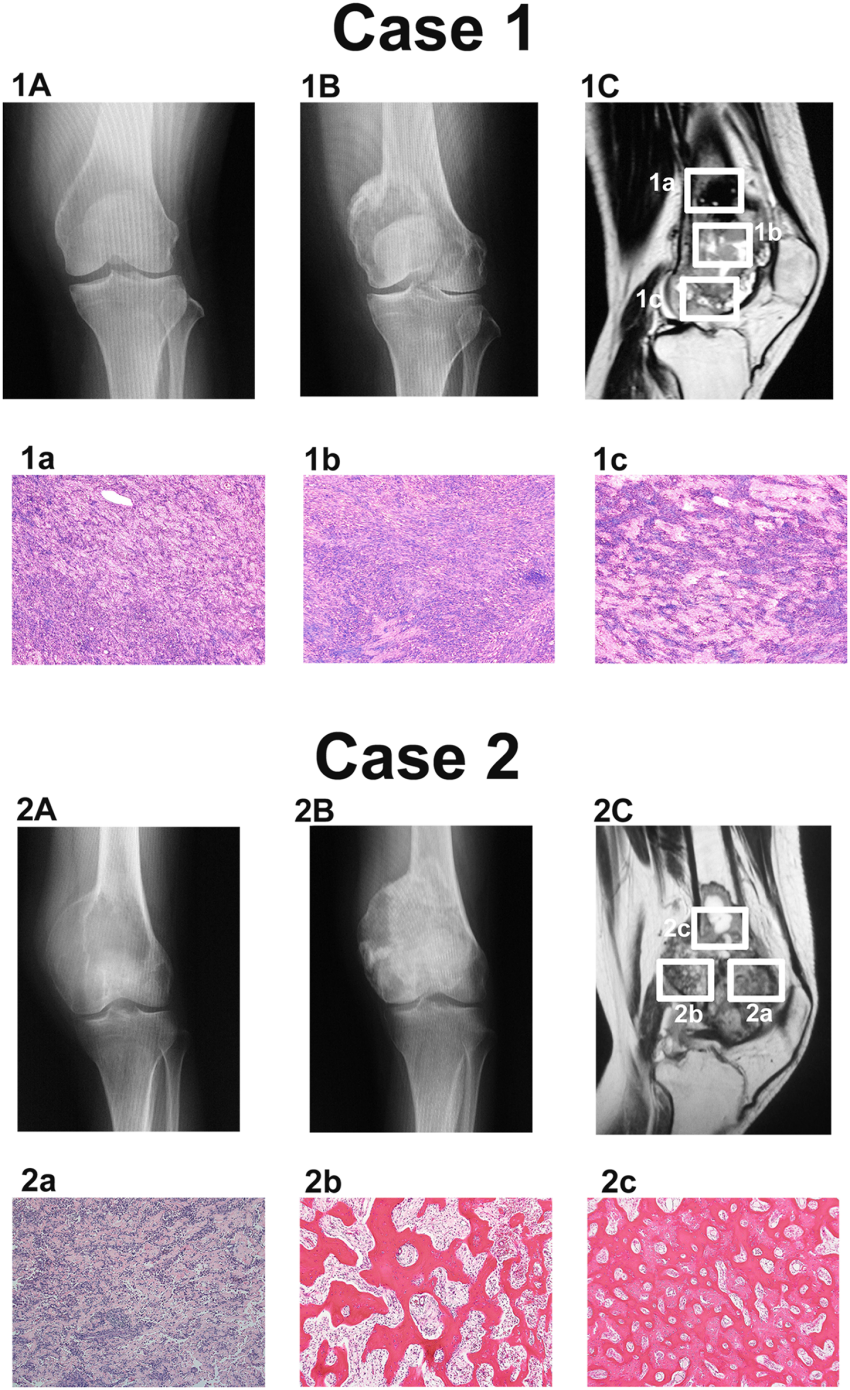
Radiography, magnetic resonance imaging and the cytological evaluation of the samples in the test set. The numbers in the panels refer to case numbers (Table 1). Radiographs (A: pre-treatment state, B: post-treatment state) and magnetic resonance imaging (C: post-treatment state) demonstrating GCTB in the knee. The tumor is located in the intramedullary space of distal femur. a, b and c, The typical microscopic appearance of the tumor mass lesion (H&E staining, 100×). In case 1: (1a) proximal lesion, (1b) intermediate lesion, (1c) distal lesion, (each surrounded by a white square). In case 2: (2a) anterior lesion, (2b) posterior lesion, (2c) proximal lesion (each surrounded by a white square).

All samples were cross-referenced with the image findings and histological features. Histologically, we found that 8 out of 10 post-treatment samples consisted of stromal cells with a few multinucleated giant cells (cases 1 and 2; Figs 1a, 1b, 1c and 2, 2a) in all four cases, while 2 post-treatment samples from case 2 seemed to contain small amounts of tumor cells in the background of fibro-osseous tissue; these 2 samples were therefore excluded from the proteomic analysis (case 2; Fig 2, 2b and 2c). Proteins were extracted from these samples for the proteomic analysis. We also performed an immunohistochemical analysis of these 4 cases, together with another 35 primary GCTB cases using formalin fixed paraffin embedded (FFPE) samples. This project was approved by the ethical review board of Juntendo University, and written informed consent was obtained from the two patients whose samples were sent for the proteomic analysis.

### iTRAQ sample labeling, mass spectrometry analysis, and peptide identification

We analyzed the proteins using isobaric tags for the relative and absolute quantification (iTRAQ) analysis; chemical labeling was performed by mass spectrometry, as described previously [15, 16]. The cell lysate samples were concentrated and buffer exchanged using cut-off spin concentrators with a molecular weight of 3.5 kDa (Tomy Seiko Co., Ltd., Tokyo, Japan), then digested for 24 h with 10 μg L-1-(4-tosylamido)-2-phenylethyl tosylphenylalanyl chloromethyl ketone—treated trypsin. Each peptide solution was labelled with one of the eight iTRAQ reagents (iTRAQ reporter ions of 113, 114, 115, 116, 117, 118, 119 and 121 mass/charge ratio) according to the manufacturer's protocol (AB SCIEX, Framingham, MA, USA). The labeled peptides were pooled and fractionated by strong cation exchange, using a ChromXP C18-CL column (Eksigent parts of AB SCIEX, Dublin, California, USA), and analyzed by nano liquid chromatography in combination with tandem mass spectrometry (LC-MS/MS); nano LC-MS was performed on a TripleTOF 5600 mass spectrometer for MS/MS (AB SCIEX) which was interfaced with a nano LC system (AB SCIEX) [17]. The protein identification and relative quantification were carried out using the ProteinPilot software program (Version 4.5, AB SCIEX) [18].

The functions of the various protein contents were searched against the Swissport database (release 10/16/2013) using the search algorithm within the ProteinPilot software program and the Analyst TF software program (AB SCIEX). The protein ratios were normalized using the overall median ratio for all of the peptides in the sample for each of the ratios in all of the individual experiments.

Two independent iTRAQ experiments were carried out to profile and quantitate the proteome and 3 technical replicates were used to determine the cutoff for significant fold-changes. A confidence cutoff of >95% was applied for protein identification, and a >1.2-fold change cutoff was selected for all of the iTRAQ ratios in order to classify proteins as upregulated or downregulated as previously described [19].

### Pathway analysis

The Ingenuity Pathway Analysis software program (Ingenuity Systems, Redwood City, CA) was further used to determine the functional pathways of the identified genes. The IPA software program contains a database of biological interactions among genes and proteins, which was used to calculate the probability of relationships among each of the canonical pathways, the upstream pathways and the identified proteins. The IPA program scans the proteins that are entered by the user to identify networks using the Ingenuity Pathway Knowledge Base (IPKB) based on interactions between identified proteins and the known and hypothetical interacting genes that are stored in the program. We have licensed the Ingenuity Pathway Analysis System (IPA; Qiagen, Redwood City, CA, USA) for the free use since 2013.

### Gelatin zymography

The one protein that was identified by the proteomic analysis was further studied by zymography. The proteolytic activity of MMP-9 in the GCTB samples was examined by gelatin zymography using a Gelatin-zymography Kit (Cosmo-Bio, Tokyo, Japan) according to the manufacturer’s instructions. Total protein lysate (15 μg) (similar to that in which protein samples were prepared for the i-TRAQ analysis) was subjected to electrophoresis on polyacrylamide SDS gels containing gelatin. The gel was washed and incubated for 24 hours at 37°C in the reaction buffer. After the enzymatic reaction, the gel was stained in a staining buffer for 30 minutes at room temperature, and then destained with destaining buffer for 1 hour. A 92-kDa proteolytic band, which corresponded to the active form of MMP-9, was scanned using a photo scanner. For the semi-quantitation of the gelatinase activity, the band was analyzed using the Multi Gauge version 3.0 software program (Fuji Film, Tokyo, Japan).

### Immunohistochemistry

An immunohistochemical analysis was performed using the available FFPE GCTB samples. A total of 37 cases of GCTB (2 cases that were treated with denosumab and 35 cases that were not treated with Denosumab) were analyzed. In brief, 4-μm thick tissues were autoclaved in Tris-EDTA buffer (pH 9.0) at 100°C for 30 min and incubated with a commercial monoclonal antibody against MMP-9 (dilution 1:100, NB110-57223, Novus Biologicals, Littleton, CO. USA). Immunostaining was performed using an Envison Dual Link system (DAKO). The immunohistochemically-stained sections were evaluated as described previously [20]. Immunostaining was separately scored in both mononuclear cells and multinucleated giant cells according to the following system: score 0 (no positive cells), score 1 (<5% positive cells), score 2 (5–20% positive cells), score 3 (20–50% positive cells), score 4 (50–80% positive cells) and score 5 (>80% positive cells). The immunohistochemical scoring of MMP-9 was independently evaluated by 2 of the authors (T.S., who is a board certified pathologist, and K.M.) without prior knowledge of the clinicopathological data. Discrepancies were resolved through re-evaluation until the authors reached a consensus.

## Results

### The identification of differentially expressed proteins associated with denosumab treatment in GCTB

To identify the protein expression profiles that were associated with denosumab treatment, we performed a comparative proteomic analysis using samples that were obtained before denosumab treatment (pre-Tx) and after denosumab treatment (post-Tx). We designed this study, which consisted of two profile sets (the test set [cases 1 and 2]; and the validation set [cases 3 and 4]) to accurately identify the protein profiles in GCTB that are affected by denosumab. The twelve samples that were used for the proteomic studies were as follows: the test set included 1 pre-Tx sample and 3 post-Tx samples from case 1, and 1 pre-Tx sample and 1 post-Tx sample from case 2; the validation set included 1 pre-Tx sample and 2 post-Tx samples from case 3, and 1 pre-Tx sample and 2 post-Tx samples from case 4. These histological findings were validated by an experienced board-certified pathologist (TS) in our institution.

We performed i-TRAQ and identified approximately 1500–2200 proteins in each analysis (data not shown). In the test set, we finally acquired 4 protein profiles that were differentially expressed in pre-Tx and post-Tx samples: (A. pre-Tx vs. post-Tx A [case 1]; B. pre-Tx vs. post-Tx B [case 1], C. pre-Tx vs. post-Tx C [case 1]; D. pre-Tx vs. post-Tx D [case 2]). As a validation set, we obtained 4 additional protein profiles that were differentially expressed in the pre-Tx and post-Tx samples: (E. pre-Tx vs. post-Tx E [case 3]; F. pre-Tx vs. post-Tx F [case 3]; G. pre-Tx vs. post-Tx G [case 4] and H. pre-Tx vs. post-Tx H [case 4]). We found that 263, 281, 334, 174, 180, 212, 222 and 225 proteins were significantly and differentially expressed in profiles A (p < 0.05; S1 Table), B (p < 0.05, S2 Table), C (p < 0.05, S3 Table), D (p < 0.05, S4 Table), E (p < 0.05, S5 Table), F (p < 0.05, S6 Table), G (p < 0.05, S7 Table) and H (p < 0.05, S8 Table), respectively. Among the 4 protein profiles in the test set (profiles A-D), there were 69 proteins that were consistently differentially expressed (32 were downregulated and 37 were upregulated) in the post-Tx samples in comparison to the pre-Tx sample (S9 Table from cases 1 and 2). Among the 4 protein profiles in the validation set (profiles E-H), we identified 76 proteins that were consistently differentially expressed (44 were downregulated and 32 were upregulated) in the protein comparisons between the pre-Tx and post-Tx samples (S10 Table from cases 3 and 4). Finally, in a combined analysis, which included the test set (cases 1 and 2) and the validation set (cases 3 and 4), among the 8 protein profiles, there 32 proteins were consistently differentially expressed (13 were downregulated and 19 were upregulated) in the post-Tx samples in comparison to the pre-Tx samples (Table 2). The functional classification of the proteins using GO terms revealed that they that were related to extracellular matrix organization, immune response, regulation of apoptosis, transmembrane transport, oxidation reduction, cellular homeostasis, protein localization, translation, cell motion, defense response, skeletal system development, protein folding and other functions (Table 2). Through our systematic analyses, which included the test set and the validation set, the 4 proteins (among the proteins that were consistently identified) that were most overexpressed in the post-Tx samples in comparison to the pre-Tx samples were serum albumin (ALBU), lumican (LUM), Ig kappa chain C region (IGKC), and Ig gamma-1 chain C region (IGHG1). The 4 most underexpressed proteins in the post-treatment samples were tartrate-resistant acid phosphatase type 5 (ACP5), carbonic anhydrase 2 (CAH2), matrix metalloproteinase-9 (MMP-9), and creatine kinase B-type (KCRB). Among these significantly upregulated and downregulated proteins, LUM, ACP5, MMP-9 and CAH2 have been reported to be functionally involved in bone metabolism in GCTB [3, 8, 21].

**Table 2:** Comparative protein expression list of GCTB samples between pre-Tx and post-Tx with denosumab.

Accession no.	Symbol	Protein name	Fold difference Avg.	P value Avg.	Functional annotation
P01860	IGHG3	Ig gamma-3 chain C region	2.06	1.53E-02	immune response
P13796	PLSL	Plastin-2	-1.63	1.56E-03	immune response
P01834	IGKC	Ig kappa chain C region	2.8	1.28E-02	immune response
P01024	CO3	Complement C3	1.66	8.33E-05	immune response
P51884	LUM	Lumican	3.41	0.00E+00	extracellular matrix organization
P50454	SERPH	Serpin H1	-1.64	7.92E-04	extracellular matrix organization
P14780	MMP9	Matrix metalloproteinase-9	-3.52	6.50E-04	extracellular matrix organization
P99999	CYC	Cytochrome c	-1.82	4.02E-03	regulation of apoptosis
P14625	ENPL	Endoplasmin	-1.31	6.54E-03	regulation of apoptosis
P04080	CYTB	Cystatin-B	-2.47	7.58E-04	regulation of apoptosis
P21281	VATB2	V-type proton ATPase subunit B, brain isoform	-2.28	8.33E-05	transmembrane transport
P38606	VATA	V-type proton ATPase catalytic subunit A	-1.97	1.74E-03	transmembrane transport
P36543	VATE1	V-type proton ATPase subunit E 1	-2.11	1.67E-03	transmembrane transport
P48735	IDHP	Isocitrate dehydrogenase [NADP], mitochondrial	-1.49	2.58E-03	oxidation reduction
P40939	ECHA	Trifunctional enzyme subunit alpha, mitochondrial	-1.42	4.50E-04	oxidation reduction
P04406	G3P	Glyceraldehyde-3-phosphate dehydrogenase	-1.49	3.50E-03	oxidation reduction
P02790	HEMO	Hemopexin	2.1	1.73E-03	cellular homeostasis
P02787	TRFE	Serotransferrin	2.61	0.00E+00	cellular homeostasis
P12277	KCRB	Creatine kinase B-type	-5.92	0.00E+00	cellular homeostasis
P21333	FLNA	Filamin-A	1.38	5.00E-05	protein localization
P13639	EF2	Elongation factor 2	-1.3	1.34E-03	translation
P06753-2	TPM3	Isoform 2 of Tropomyosin alpha-3 chain	1.39	3.31E-03	cell motion
P01009	A1AT	Alpha-1-antitrypsin	2.23	0.00E+00	defense response
P13686	PPA5	Tartrate-resistant acid phosphatase type 5	-3.55	1.14E-02	skeletal system development
P49368	TCPG	T-complex protein 1 subunit gamma	-1.34	7.95E-03	protein folding
P00918	CAH2	Carbonic anhydrase 2	-3.88	2.50E-05	other
Q99798	ACON	Aconitate hydratase, mitochondrial	-1.52	2.17E-04	other
Q15293	RCN1	Reticulocalbin-1	-1.38	1.31E-02	other
Q09666	AHNK	Neuroblast differentiation-associated protein AHNAK	1.5	5.38E-03	other
P02768	ALBU	Serum albumin	4.42	0.00E+00	other
P02545	LMNA	Prelamin-A/C	2.06	7.50E-05	other
P01857	IGHG1	Ig gamma-1 chain C region	2.89	7.28E-03	other

Accession numbers of proteins were derived from Swiss-Plot data base.

Functional annotations are classified using Gene ontology terms.

### Network analyses of obtained expression profiles using IPA

**Fig 3:**
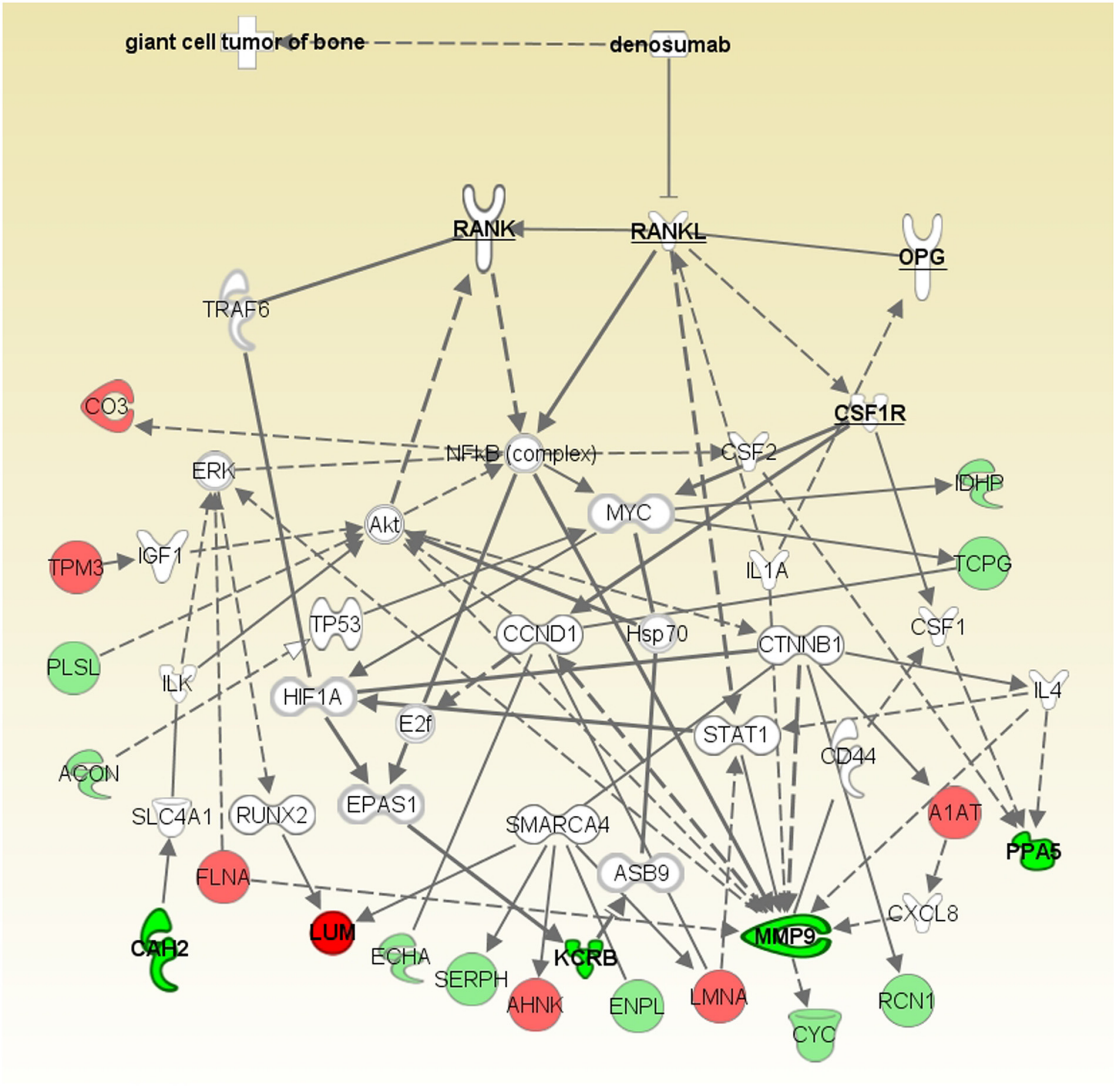
The hypothesized pathway of the effects of denosumab in GCTB based on the data from our proteomic profile. Each protein within the network is shown as a node. The lines indicate the interactions between the nodes. The nodes that were identified as being upregulated or downregulated in the proteomic profiles are indicated in red or green, respectively.

In order to further understand the key signaling networks that are associated with denosumab treatment, protein expression profiles were subjected to an IPA (Ingenuity Systems, Inc, CA, USA). The software program was used to construct shortest path networks to connect the most upregulated and downregulated proteins and the proteins that are involved in the RANK/RANKL pathway using the GeneGo global database of established canonical pathways. The proteins that were identified in the iTRAQ analysis. Color was used to indicate the direction of change in fold expression: upregulated proteins are indicated in red, while downregulated proteins are indicated in green (Fig 3). The network analyses identified several subpathways, including the matrix metalloprotease pathway (data not shown).

### The protein expression and proteolytic activity of MMP-9

**Fig 4:**
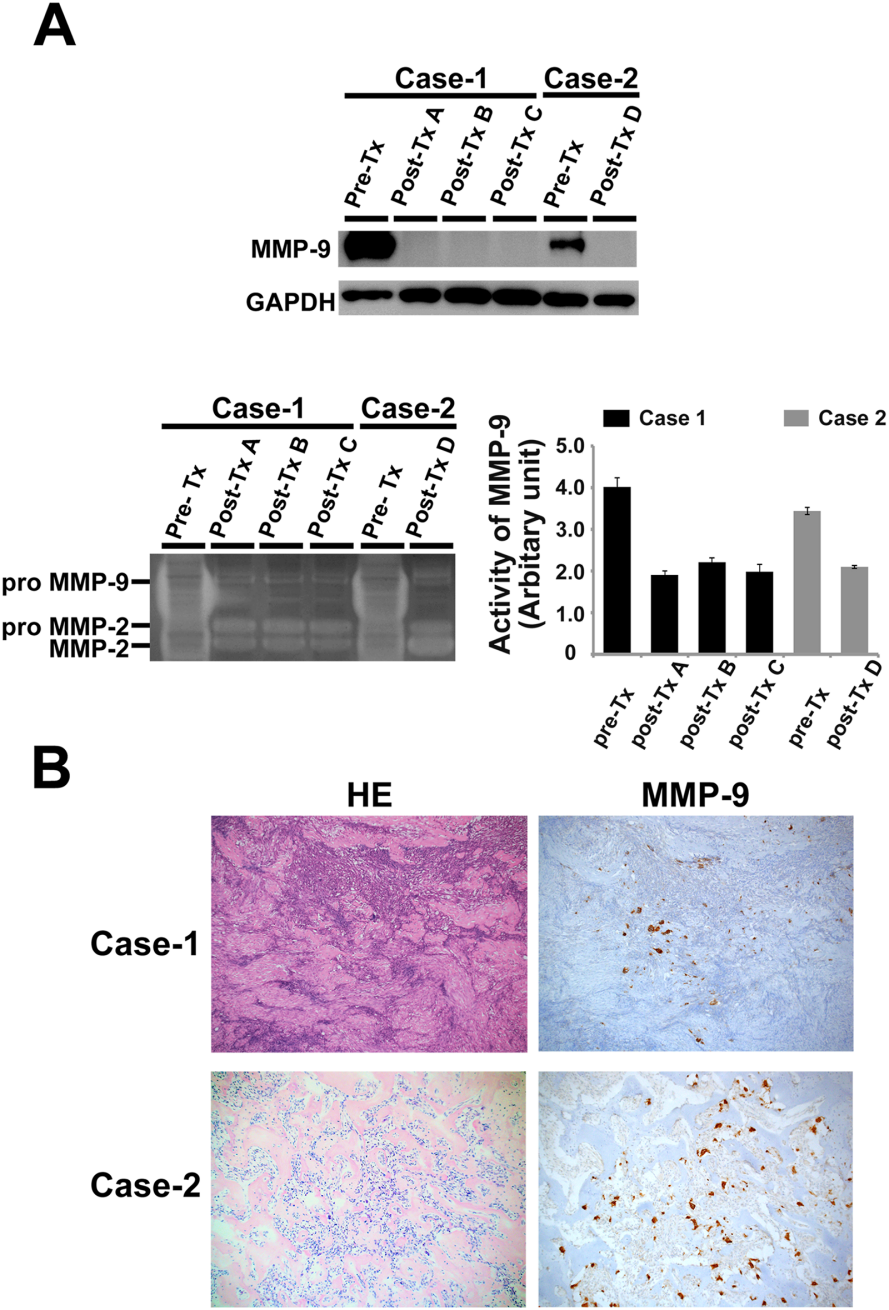
A validation study on the expression and activity of MMP-9 in the test set. (A) Western blot analysis showed that treatment with denosumab resulted in a marked decreased in the expression levels of MMP-9 in cases 1 and 2 (upper panel). A zymogram indicating that gelatinolytic activity is decreased in post-Tx samples in comparison to pre-Tx samples in cases 1 and 2 (lower left panel). The bar graph shows the quantitative results of zymography (lower right panel). (B) In both the post-Tx samples (cases 1 and 2), HE stain showed that the reduction of osteoclast-like giant cell numbers and the replacement of tumor cells were by the fibroosseous tissue (left panels). In both the post-Tx samples (cases 1 and 2), the immunohistochemical analyses showed the expression and localization of MMP-9 in both stromal cells and osteoclast giant cells (right panels).

**Fig 5:**
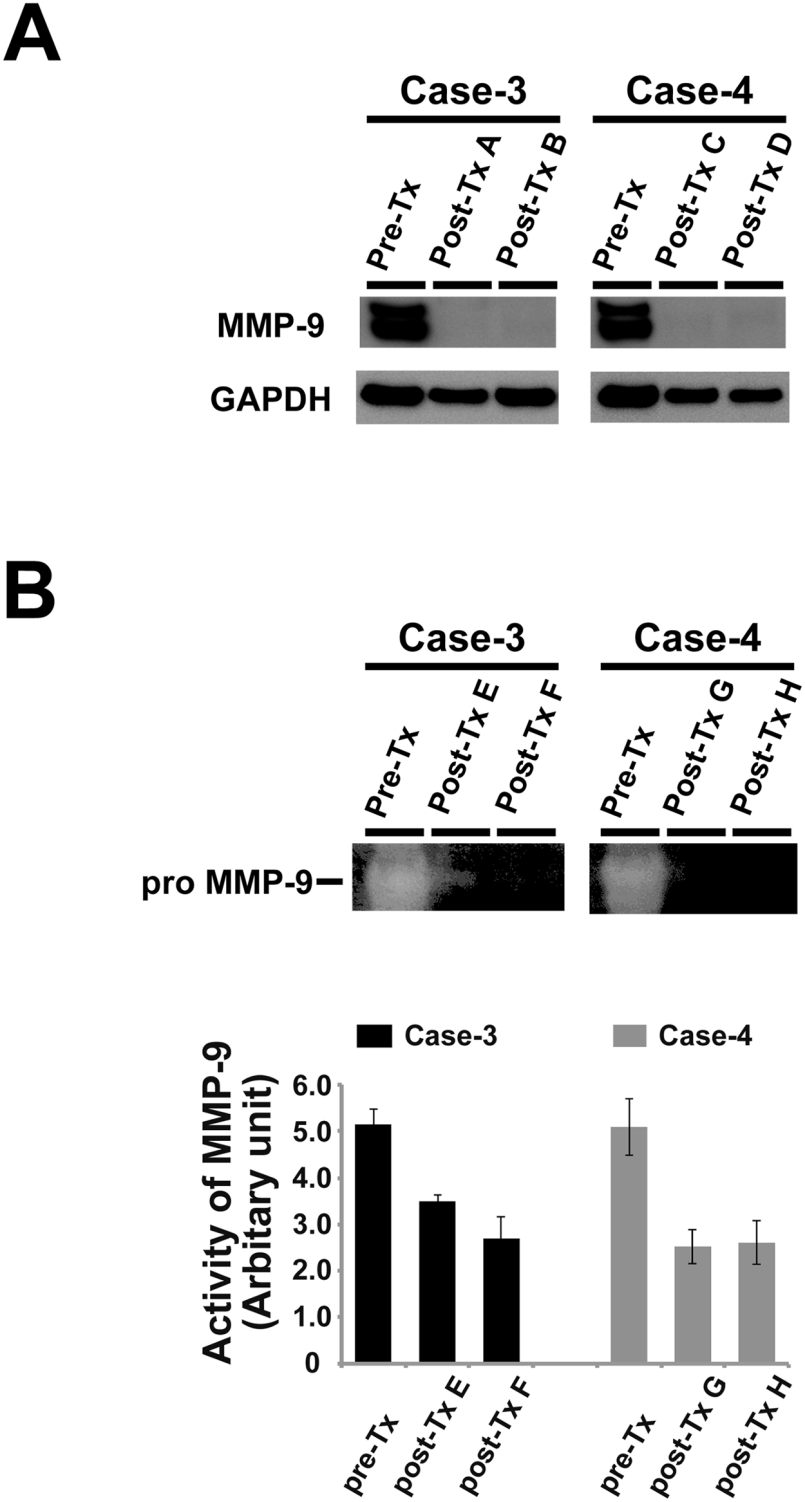
A validation study on the expression and activity of MMP-9 in the validation set. (A) In the Western blot analysis, the expression levels of MMP-9 were found to decrease in the post-Tx samples in comparison to the pre-Tx expression levels in cases 3 and 4. (B) In the zymographic analysis, the gelatinolytic activity of MMP-9 was decreased in the post-Tx samples in comparison to the pre-Tx samples in cases 3 and 4 (upper panel). The bar graph shows the quantitative results of the zymographic analysis (lower panel).

Based on our proteomic profile and the IPA, MMP-9 was revealed to be the most downregulated protein in the post-Tx samples. *In vitro* assays were performed to validate the consistency of our proteomic profile data. The GCTB samples were subjected to Western blotting. The results showed that MMP-9 expression was markedly decreased in the post-Tx samples in comparison to the pre-Tx samples in all 4 cases, including the test set and the validation set (Fig 4A [upper panel] and Fig 5A). A zymographic analysis using the GCTB samples was performed to evaluate MMP-9 activity because zymography is capable of detecting the active forms of MMP-9. MMP-9 activity was highest in the pre-Tx samples of each case, and was markedly decreased in the post-Tx samples of each case (Fig 4A [lower panel] and Fig 5B). These results, which were consistent with the results of the proteomic analysis and the IPA data, suggest that denosumab treatment decreased the expression and activity of MMP-9 in GCTB.

### The localized expression of MMP-9 in the denosumab-treated samples as determined by immunohistochemistry

The localization of MMP-9 was examined by immunohistochemistry in the post-Tx samples that were used for the i-TRAQ analyses (Fig 4B). The nuclear and cytoplasmic staining of MMP-9 was found in both stromal cells and osteoclastic giant cells. Only a small number of osteoclastic giant cells remained in the denosumab-treated samples; however, the residual osteoclastic giant cells strongly expressed MMP-9 (score 5), the staining intensity of which did not change in the pre- and post-treatment samples of either case (data not shown). The staining scores of the stromal cells decreased from score 3 (pre-Tx samples) to score 2 (post-Tx samples) in our series (data not shown).

### The association between MMP9 expression, as determined by immunohistochemistry, and the clinical characteristics

We performed an immunohistochemical analysis to evaluate the correlation between the clinicopathologic factors and MMP-9 expression in an additional 35 primary GCTB patients The staining scores of the patients were as follows: score 1 (n = 5), score 2 (n = 19), and score 3 (n = 11) (κ = 0.839, S11 Table). The clinicopathological parameters included age (median: 32 years), sex, location and surgical treatment. None of these parameters was significantly correlated with the MMP-9 expression level (Chi-squared test; P > 0.05) (S12 Table). Furthermore, the survival analysis revealed that the expression of MMP-9 did not significantly affect recurrence or metastasis in GCTB patients (log rank test; P > 0.05) (data not shown).

## Discussion

The therapeutic effect of denosumab against GCTB depends on the inhibition of osteoclast activity through the RANK/RANKL pathway [22]. To date, several *in vitro* studies of GCTB have been performed using cell lines. However, the repeated passaging of cultures might induce biological and functional alterations in the original character of the stromal cells, including a gradual loss in the ability of the stromal cells to induce osteoclast-like giant cells when co-cultured with osteoclast precursors [5, 23]. Furthermore, it has been shown that denosumab does not exhibit an inhibitory effect against the growth of stromal cells or regulate RANKL, OPG or M-CSF expression at either the mRNA or the protein level [24]. In contrast, recent clinical studies have demonstrated that denosumab treatment reduced the relative content of the neoplastic stromal cells in patients with GCT [12], and another immunohistochemical study using tissue specimens from patients with GCTB indicated that the overexpression of RANKL by tumor stromal cells may be responsible for the osteolytic behavior of these cells in GCTBs [4]. However, these data have not necessarily provided an appropriate biological model that reflects the molecular dynamics in GCTB. In order to understand the molecular biology of denosumab treatment in GCTB, it seems to be necessary to employ a different experimental approach using clinical samples.

In the present study, in which in surgical specimen were obtained from GCTB patients who were treated with denosumab, the comparative proteomic analysis revealed 32 proteins that were differentially expressed after treatment with denosumab; 13 of these were upregulated and 19 were downregulated. Although several proteins were identified as most upregulated in the denosumab-treated samples might have been affected by the serum, it would be of interest to focus on Lumican (LUM), which was overexpressed with more than a 3-fold difference. LUM, which is member of the small leucine-rich proteoglycan (SLRP) family, has a functional role in the extracellular matrix; however, it has not been extensively studied [3, 25, 26]. Interestingly, a recent study demonstrated a potential role of LUM as biomarker of aggressive behavior in GCTB. LUM was expressed at a lower level in a group of GCTB patients with lung metastases in comparison to non-metastatic patients [3]. This finding supports the therapeutic effect of denosumab in GCTB; though it is not clear whether denosumab treatment can also suppress lung metastasis in patients with GCTB.

With regard to the downregulated proteins, creatine kinase type B (KCRB), which is also known as CKB, plays an important role in maintaining cellular homeostasis [27]. CKB expression has been shown to be greatly increased during osteoclastogenesis, while the blocking of CKB has been demonstrated to reduce bone resorption by human osteoclasts *in vitro* [28]. Thus, CKB has a crucial role in osteoclast-mediated bone resorption. In addition, carbonic anhydrase 2 (CAH2) has been shown to mediate the production of hydrogen ions (H+) from CO_2_ and H_2_O in the cytoplasm. Mature osteoclasts secrete H+ extracellularly through H+-ATPase and several proteases, such as cathepsin K and matrix metalloproteinase-9 (MMP-9) [8, 29]. CAH2 is indispensable in the resorptive process of bone, and osteoclasts which lack CAH2 or H+-ATPase are incapable of acidifying the resorptive bone microenvironment [30]. PPA5, which is also known as TRACP (Tartrate-resistant acid phosphatase), is an iron-containing enzyme that is highly expressed in osteoclasts. Recent data have demonstrated the clinical utility of TRACP as a marker of bone resorption [31]. TRACP is also used in immunohistochemistry as a marker of osteoclast differentiation [32]. MMP-9 (matrix metalloproteinases-9) is one of the MMPs, a family of the prominent enzymes that degrade different components of the extracellular matrix [33]. Among the various MMPs, MMP-2 (gelatinase A) and MMP-9 (gelatinase B) may play a significant role in tumor progression and invasion in GCT [20, 34]. Bone matrix destruction via type-I collagen degradation by osteoclasts leaves behind denatured type-I collagen (gelatin), which MMP-2 and MMP-9 degrade, leading to osteolysis [35, 36]. A previous study implied that MMP-9 is indirectly responsible for the osteolysis via transcriptional activation by RANKL signals through TRAF6 and that NFATc1 is a downstream effector of RANKL signaling to modulate *MMP-9* gene expression during osteoclast differentiation [37].

The IPA software program was used to allow a better understanding of our proteomic data. It identified a significant biological network that was related to denosumab treatment in GCTB. The pathway analysis indicated molecular interactions of identified proteins including the 5 most dysregulated proteins (LUM, KCRB, CAH2, PPA5 and MMP9) and the interaction of proteins in the RANK/RANKL pathway (Fig 3). The pathway analysis also identified several subpathways that were affected, to a statistically significant extent, in the GCTB patients—including the inhibition of the matrix metalloproteases pathway (data not shown). A number of studies have shown that MMPs are also synthesized in tumor cells to regulate tumor invasion and metastasis in malignant tumors [34, 38, 39]. MMP1 and a disintegrin-like metalloproteinase with thrombospondin motifs 1 (ADAMTS1), shed epidermal growth factor (EGF)-like ligands to suppress OPG, indirectly leading to osteoclastogenesis [40]. Moreover, MMP13 can activate MMP9 and TGFβ to increase the local expression of RANKL at the tumor-bone interface in breast cancer [41, 42]. The MMPs have also been shown to be associated with the local aggressive behavior of GCTB [20, 34, 43]. MMP-9 was therefore presumed to play an important role in this subpathway in GCTB. We further investigated both the activity and expression of MMP-9 to clarify their association with denosumab treatment in GCTB. Using zymography, we confirmed that the activity of MMP-9 was decreased in post-Tx samples in comparison to pre-Tx samples, indicating that denosumab inhibited the gelatinolytic activity of MMP-9 through a certain mechanism. We were also able to confirm MMP-9 expression in both osteoclast-like giant cells and stromal cells by immunohistochemistry. These results are consistent with previous studies that showed that two of the cellular components of GCTB expressed MMP-9 [44]. The expression level of MMP-9 in stromal cells was slightly decreased in post-Tx samples in comparison to pre-Tx samples, but not in osteoclast-like giant cells. This result suggests that the MMP-9 expression in stromal cells also has an important role in bone destructions, which is consistent with previous findings [43]. Furthermore, although the reduction of osteoclast-like giant cell numbers and the replacement of tumor cells were by the fibro-osseous tissue have been shown to be the therapeutic effects of denosumab, these findings suggest that even residual tumor tissue (composed of only stromal cells) can destroy bone, and that the therapeutic application of denosumab would still be necessary in the treatment of these lesions.

Based on these findings, we hypothesized that the MMP-9 expression level in GCTB might affect the local recurrence. We investigated the correlation between clinicopathological factors (age, sex, location and surgical procedure) and MMP-9 expression in 35 primary GCTB samples; however, none of these factors was found to be correlated with MMP-9 expression. Furthermore, the expression level of MMP-9 was not associated with local recurrence or metastasis in the 35 primary GCTB samples from patients who did not receive denosumab treatment (data not shown). However, the immunohistochemical detection of MMP-9 might be inappropriate for examining the physiological activity of MMP-9. MMP-9 is secreted extracellularly in an inactive form; MMP-9 subsequently changes to the active form after the cleavage of its propeptide. Thus, quantitative change in the level of MMP-9 that is detected by immunohistochemistry does not necessarily reflect the level of protease activity. Although the prognostic value of MMP-9 was not well explored in the present study, the change in MMP-9 expression that was observed after denosumab treatment was an interesting finding which deserves further investigation.

In summary, we demonstrated the first proteome expression analysis of GCTB treated with denosumab using clinical samples. The proteomic comparison identified a number of differentially expressed proteins. These proteins may reflect the molecular interactions of bone metabolism in GCTB. The expression patterns of these proteins may help to explain the unknown mechanism behind the anti-osteolytic effect of denosumab in GCTB. Further investigation may reveal potent proteins that are involved in the pathophysiology of GCTB. A large-scale study with more detailed investigative techniques will be required to increase the reliability of our data, and further clarify the biological mechanisms behind the effects of denosumab in GCTB.

## Supporting Information

S1 Table Protein expression profiling of A. Comparative protein expression list of GCTB samples between pre-TX vs. post-Tx A.
(XLSX)

S2 Table Protein expression profiling of B. Comparative protein expression list of GCTB samples between pre-TX vs. post-Tx B.
(XLSX)

S3 Table Protein expression profiling of C. Comparative protein expression list of GCTB samples between pre-TX vs. post-Tx C.
(XLSX)

S4 Table Protein expression profiling of D. Comparative protein expression list of GCTB samples between pre-TX vs. post-Tx D.
(XLSX)

S5 Table Protein expression profiling of E. Comparative protein expression list of GCTB samples between pre-TX vs. post-Tx E.
(XLSX)

S6 Table Protein expression profiling of F. Comparative protein expression list of GCTB samples between pre-TX vs. post-Tx F.
(XLSX)

S7 Table Protein expression profiling of G. Comparative protein expression list of GCTB samples between pre-TX vs. post-Tx G.
(XLSX)

S8 Table Protein expression profiling of H. Comparative protein expression list of GCTB samples between pre-TX vs. post-Tx H.
(XLSX)

S9 Table The combined protein expression profiling of the test set. Comparative protein expression list of GCTB samples between pre-TX vs. post-Tx in cases 1 and 2.
(XLSX)

S10 Table The combined protein expression profiling of the validation set. Comparative protein expression list of GCTB samples between pre-TX vs. post-Tx in cases 3 and 4.
(XLSX)

S11 Table The clinicopathological characteristics of additional 35 GCTB patients. (XLSX)

S12 Table The correlation between MMP-9 expression and clinicopathological features in additional 35 GCTB patients. (XLSX)
